# Supplementary material for: Silicon and oxygen synergistic effects for the discovery of new high-performance nonfullerene acceptors
Source: Nat Commun. 2020 Nov 16;11:5814. doi: 10.1038/s41467-020-19605-z (PMC7669892; doi:10.1038/s41467-020-19605-z)
Supplement: Supplementary file 1 — Supplementary Information [file 41467_2020_19605_MOESM1_ESM.pdf]

Supplementary Information

Silicon and Oxygen Synergistic Effects for Discovery of New High-Performance Nonfullerene Acceptors

Ying Qin[1], Hui Chen[2], Jia Yao[3], Yue Zhou[1], Yongjoon Cho[4], Yulin Zhu[2], Beibei Qiu[5], Cheng-Wei Ju[1], Zhi-Guo Zhang[3*], Feng He[2*], Changduk Yang[4], Yongfang Li[5] and Dongbing Zhao[1*]

[1] State Key Laboratory and Institute of Elemento-Organic Chemistry, College of Chemistry, Nankai University, Tianjin, 300071, China
[2] Shenzhen Grubbs Institute and Department of Chemistry, Southern University of Science and Technology, Shenzhen 518055, China
[3] State Key Laboratory of Chemical Resource Engineering, Beijing Advanced Innovation Center for Soft Matter Science and Engineering, Beijing University of Chemical Technology, Beijing, 100029, China
[4] Department of Energy Engineering, School of Energy and Chemical Engineering, Perovtronics Research Center, Low Dimensional Carbon Materials Center, Ulsan National Institute of Science and Technology (UNIST), Ulsan 44919, Republic of Korea
[5] Beijing National Laboratory for Molecular Sciences, CAS Key Laboratory of Organic Solids, Institute of Chemistry, Chinese Academy of Sciences, Beijing 100190, China
Ying Qin and Hui Chen contributed equally to this work.
*Corresponding authors: D. Z. (dongbing.chem@nankai.edu.cn), F. H. (hef@sustech.edu.cn), Z.-G. Z. (zgzhang@mail.buct.edu.cn)

1. Supplementary Methods

Materials and Instrumentation Methods

All reactions were set up using standard Schlenk techniques and carried out under a N_2 atmosphere with dry solvents. Commercially available chemicals were obtained from Infinity Scientific, Heowns, Admas, Alfa Aesar, J&K, Sigma-Aldrich, Energy Chemical and TCI and used as received unless otherwise stated. Analytical thin layer chromatography (TLC) was performed on silica gel 60 F_{254} glass plates. TLC plates were visualized by exposure to short wave ultraviolet light (254 nm, 365 nm) and/or iodine.

UV-Vis was recorded with SPECORD® 210 PLUS spectrometers. To reduce the fluctuation in the excitation intensity, the lamp was kept on for 1 hour prior to the experiment. Cyclicvoltammetry (CV) was performed with a CHI660E potentiostat. All measurements were carried out in a one-compartment cell under a nitrogen atmosphere, equipped with a glassy-carbon electrode, a platinumcounter-electrode, and an Ag/Ag^+ reference electrode with a scan rate of 100 mV s^{-1}. The supporting electrolyte was a 0.1 mol/L acetonitrile solution of tetrabutylammonium hexafluorophosphate. All potentials were corrected against Fc/Fc^+.

Nuclear magnetic resonance (NMR) spectra were recorded on Bruker AV 400 spectrometer at 400MHz (^{1}H NMR), 100MHz (^{13}C NMR) and 376MHz (^{19}F NMR) using $CDCl_3$ as solvent. Proton and carbon chemical shifts are reported relative to the solvent used as an internal reference ($CDCl_3$: δ_H = 7.26 ppm, δ_C = 77.16 ppm). High resolution mass spectra (HRMS) of product were recorded on Varian 7.0T FTMS with ESI resource or Bruker Autoflex III TOF/TOF.

Synthesis Procedures

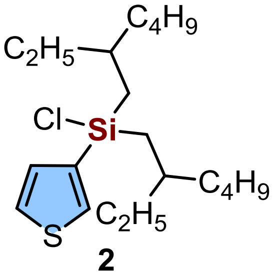

2

Under the N_2 atmosphere, ^{n}BuLi (1.6 M in hexanes; 40.0 mmol) was added dropwise to a stirred solution of 3-bromothiophene **1** (3.7 mL, 40 mmol, 1.0 eq.) in THF (100 mL) at -78 ℃ and stirred for 30 minutes at the same temperature. And then dichlorobis(2-ethylhexyl)silane (13 g, 40 mmol, 1.0 eq.) was added dropwise to the solution at -78 ℃. After addition, the mixture was slowly warmed up to room temperature and stirred overnight. The reaction was quenched by slowly adding water (1 mL) and brine (100 mL) at room temperature. The organic phase was then separated and the aqueous phase was extracted with Et_2O (2 × 100 mL). The combined organic phase was dried (Na_2SO_4) and concentrated under vacuum to give the crude product as pale yellow oil, which was used directly for the next step without further purification.

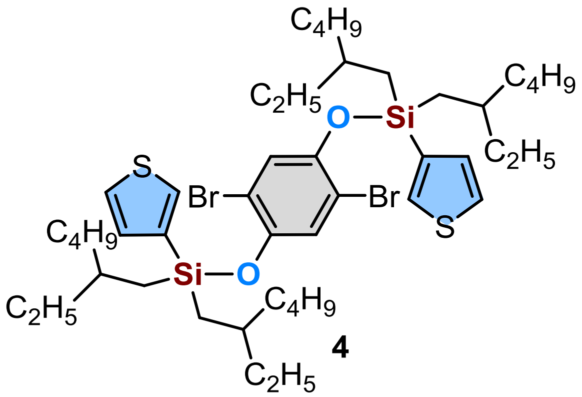

4

Under the N_2 atmosphere, the corresponding silane **2** was added dropwise to a solution of 2,5-dibromobenzene-1,4-diol **3** (5.36 g, 20 mmol, 1.0 eq.) and imidazole (4.1 g, 60 mmol, 3 eq.) in DCM (100 mL) at 25 ℃ and stirred for 30 min. And then the reaction was refluxed at 50 ℃ for additional 12 h. Then the reaction mixture was cooled down to room temperature and extracted with DCM (2 × 100 mL). The combined organic phase was dried over Na_2SO_4 and concentrated under vacuum to give the crude product as pale yellow oil. The crude residue was further purified by silica gel column (PE:DCM = 5:1) to give the desired compound **4** (15.77 g, 84%) as colorless oil. **^{1}H NMR** (400 MHz, $CDCl_3$) δ 7.66 (d, J = 2.2 Hz, 2H), 7.40 (dd, J = 4.8, 2.6 Hz, 2H), 7.23 (dd, J = 4.8, 0.9 Hz, 2H), 6.94 – 6.84 (m, 2H), 1.55 – 1.47 (m, 4H), 1.33 – 1.09 (m, 32H), 1.07 – 0.92 (m, 8H), 0.89 – 0.68 (m, 24H).

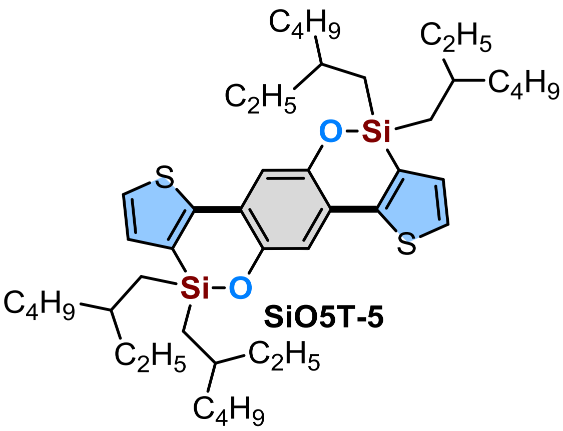

Following a modified procedure,[1] the intermediate **4** (9.41 g, 10 mmol, 1.0 eq.), $Pd(OAc)_2$ (225 mg, 1 mmol, 10 mol%), $PCy_3{\cdot}HBF_4$ (736 mg, 2 mmol, 20 mol%), Cs_2CO_3 (13 g, 40 mmol, 4.0 equiv), PivOH (306 mg, 3 mmol, 30 mol%), 3 Å MS (2.5 g, calcined before use) and the anhydrous *p*-xylene (50 mL) were added into a 100 mL flame-dried Schlenk tube with a magnetic stirring bar in a N_2 flushed glovebox. The reaction tube was capped, removed from the glovebox and the reaction mixture was stirred at 140 ℃ for 24 h. After the reaction finished, the reaction mixture was cooled down to room temperature, filtered over celite, washed with DCM, concentrated and purified by silica gel column (PE:DCM = 5:1) to give the siloxy-bridged ladder-type skeleton **SiO5T-5** (2.04 g, 26%) as yellow oil. **^{1}H NMR** (400 MHz, $CDCl_3$) δ 7.30 (d, J = 5.0 Hz, 2H), 7.15 (s, 2H), 7.05 (d, J = 5.0 Hz, 2H), 1.50 (dt, J = 14.7, 6.8 Hz, 4H), 1.31 – 1.05 (m, 32H), 1.01 – 0.68 (m, 32H). **^{13}C NMR** (101 MHz, $CDCl_3$) δ 148.85, 145.70, 130.73, 124.79, 121.82, 115.85, 35.79, 35.62, 34.89, 34.81, 29.10, 28.97, 28.84, 23.12, 22.27, 14.28, 14.26, 10.92, 10.90. **HRMS** (MALDI-TOF): calcd. for $C_{46}H_{75}O_2S_2Si_2$ $[M+H]^+$: 779.4747, Found: 779.4744.

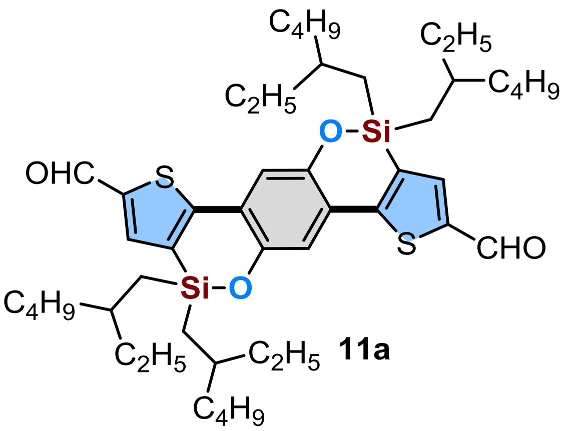

11a

A Vilsmeier reagent was prepared firstly. 20 mL of anhydrous DMF was added to a dry 100 mL two-necked round bottom flask, and the solution was cooled to 0 ℃ and stirred when 4.1 mL of phosphorous oxychloride ($POCl_3$) was added by syringe under N_2 protection. The mixture kept at 0 ℃ for 2 h, and then compound **SiO5T-5** (2.04 g, 2.62 mmol, 1.0 eq.) in dry 1,2-dichloroethane (50 mL) was added at 0 ℃ under N_2. Then, the reaction mixture was heated to 90 ℃ with stirring for 12 h. The same amount of Vilsmeier reagent were added to the reaction mixture again with continue stirring for additional 12 hours at 100 ℃. Then, the mixture was cooled down to room temperature and poured into ice water (100 mL), neutralized with Na_2CO_3, and then extracted with dichloromethane. The combined organic layer was washed with water and brine, dried over anhydrous Na_2SO_4. After removal of the solvent under reduced pressure, the residue was purified by column chromatography on silica gel using petroleum ether/dichloromethane (1:1) as eluent, yielding a yellow solid **11a** (579 mg, 26.6%). **^{1}H NMR** (400 MHz, $CDCl_3$) δ 9.94 (s, 2H), 7.72 (s, 2H), 7.22 (s, 2H), 1.50 (dt, J = 13.6, 6.4 Hz, 4H), 1.29 – 1.11 (m, 32H), 1.05 – 0.96 (m, 4H), 0.94 – 0.86 (m, 4H), 0.83 – 0.73 (m, 24H). **^{13}C NMR** (101 MHz, $CDCl_3$) δ 182.71, 156.46, 146.62, 143.36, 140.44, 133.01, 122.68, 116.72, 35.57, 35.48, 34.73, 34.63, 28.92, 28.83, 28.75, 28.65, 22.92, 21.85, 14.10, 14.07, 10.73. **HRMS** (MALDI-TOF): calcd. for $C_{48}H_{75}O_4S_2Si_2$ $[M+H]^+$: 835.4645, Found: 835.4644.

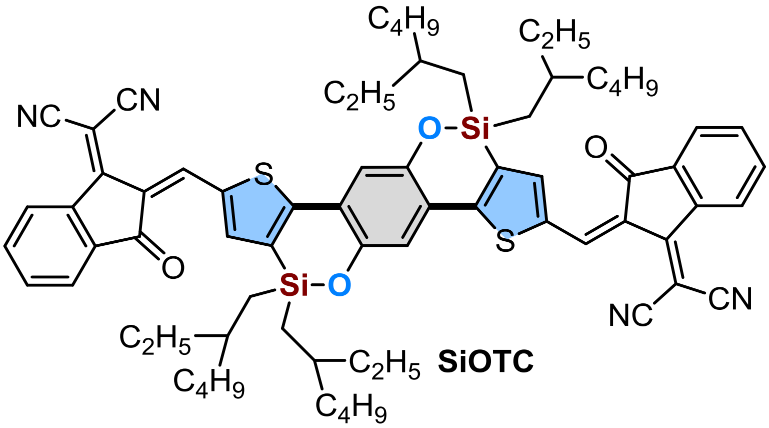

SiOTC

Following a modified literature procedure,[2] the corresponding bis-aldehyde **11a** (228 mg, 0.27 mmol) and 1,1-dicyanomethylene-3-indanone (210 mg, 1.08 mmol) were dissolved in dry $CHCl_3$ (25 mL). The mixture was deoxygenated with N_2 gas, and 1.1 mL pyridine were added and refluxed for 24 h. Then, the mixture was poured into water (100 mL) and extracted with $CHCl_3$ (2 × 100 mL). The organic layer was washed with water, and then dried over $MgSO_4$. After removing the solvent, the residue was purified using column chromatography on silica gel employing petroleum ether/$CHCl_3$ (1:2) as an eluent, yielding a dark blue solid **SiOTC** (214 mg, 66%). To ensure the device performance, the chromatographically pure **SiOTC** was further purified by re-precipitation with MeOH, and dried overnight under vacuum. **^{1}H NMR** (400 MHz, $CDCl_3$) δ 8.93 (s, 2H), 8.73 (d, J = 7.3 Hz, 2H), 7.98 (d, J = 6.1 Hz, 2H), 7.81 (q, J = 6.9, 6.4 Hz, 6H), 7.50 (s, 2H), 1.62 – 1.48 (m, 4H), 1.36 – 1.11 (m, 32H), 1.09 – 0.99 (m, 4H), 0.97 – 0.89 (m, 4H), 0.80 (q, J = 6.7 Hz, 24H). **^{13}C NMR** (101 MHz, $CDCl_3$) δ 188.40, 162.18, 160.43, 149.94, 147.61, 140.14, 137.77, 137.41, 137.08, 135.53, 134.86, 125.55, 124.04, 123.84, 123.28, 117.22, 114.56, 114.52, 70.28, 35.72, 35.69, 34.93, 34.69, 29.00, 28.91, 28.85, 23.10, 23.06, 22.27, 22.17, 22.10, 22.00, 14.28, 14.24, 10.90, 10.81. **HRMS** (MALDI-TOF): calcd. for $C_{72}H_{82}N_4O_4S_2Si_2$ [M+H]$^+$: 1187.5394, found: 1187.5392.

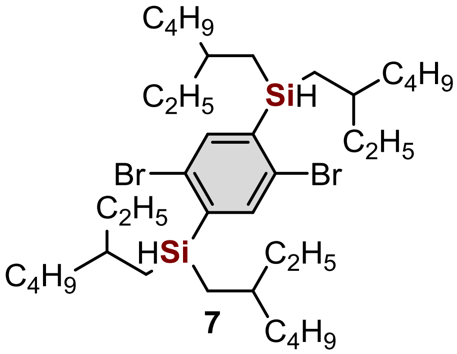

7

Chlorobis(2-ethylhexyl)silane was prepared according to the literature.[4] At −78 ℃, LDA (100 mmol) was added dropwise to the suspension of 1,4-dibromobenzene (9.32 g, 40 mmol) in THF (100 mL) containing chlorobis(2-ethylhexyl)silane (29 g). The resultant orange solution was kept at −78 °C for 2 h and then allowed to come to room temperature. After stirring overnight, the reactant was quenched by slow addition of brine (100 mL) at 0 ℃. The yellow organic phase was separated, and the water phase was extracted with ether three times. The combined organic phase was dried (Na_2SO_4) and concentrated under vacuum to give the crude product as a colorless oil. It was further purified by flash chromatography (PE) to give the desired product **7** (20 g, 67.4%) as colorless liquid.

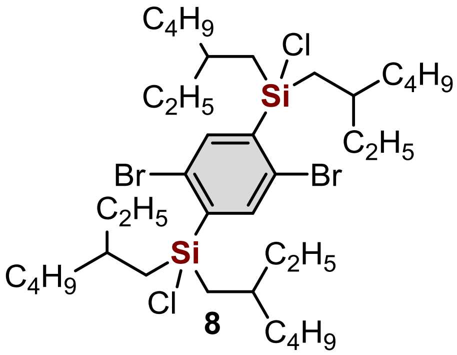

8

Following a modified literature procedure,[5] Schlenk flask was filled with dihydrosilane **7** (20 g, 26.9 mmol, 1.0 eq.) and DCM and cooled down to 0 ℃. TCCA (25 g, 107.8 mmol) was added in small portions, under intense stirring. After TCCA addition was completed, stirring was continued at 0 ℃ for 2 h, afterwards, the cooling bath was removed and the solution was allowed to come to room temperature. After stirring overnight, the mixture was filtered under vacuum and the filter residue was washed with PE. The combined solution was dried (Na_2SO_4) and concentrated under

vacuum to give the crude product **8** as pale yellow oil, which was directly used for the next step without further purification.

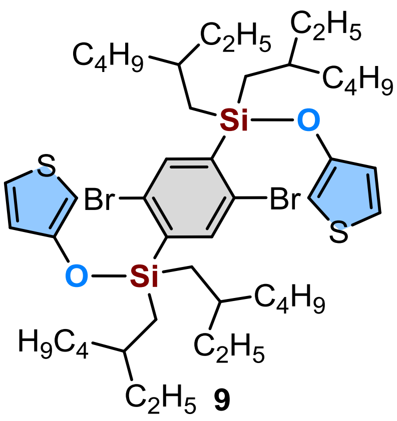

9

To an ice-cooled (0 ℃) stirred solution of the corresponding chlorosilane (1.53 mL, 6.0 mmol) in 50 mL of dry dichloromethane under N_2 was added 1H-imidazole (7.3 g, 108 mmol) and. The mixture was added a solution of thiophen-3-ol (10.8 g, 108 mmol, 4.0 eq.) in 10 mL dichloromethane followed by addition of catalytic amount of DMAP (0.5 g) in 2 mL of dichloromethane. The resulting mixture was heated to 60 ℃ and stirred for additional 2 h. After dilution with dichloromethane, the mixture was washed (brine, 3 × 20 mL), dried ($MgSO_4$), and concentrated. Flash silica gel column chromatography(PE:DCM = 5:1) purification of the residue gave the product **9** (13.46 g, 53%) as colorless oil. **^{1}H NMR** (400 MHz, $CDCl_3$) δ 7.93 (s, 2H), 7.16 (s, 2H), 6.78 (d, J = 5.1 Hz, 2H), 6.38 (s, 2H), 1.49 – 1.39 (m, 4H), 1.32 – 1.03 (m, 40H), 0.87 – 0.63 (m, 24H).

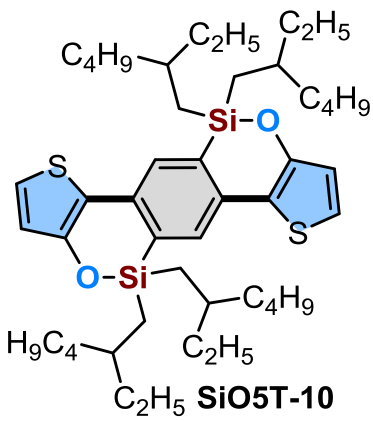

SiO5T-10

An oven dried 100 mL schlenk flask was charged with the intermediate **9** (9.47 g, 10 mmol), $Pd(OAc)_2$ (225 mg, 1 mmol, 10 mol%), PCy_3 HBF_4 (736 mg, 2 mmol, 20 mol%), Cs_2CO_3 (13 g, 40 mmol, 4.0 equiv), PivOH (306 mg, 3 mmol, 30 mol%), 3 Å MS (2.5 g, calcined before use) and anhydrous *p*-xylene (50 mL) under nitrogen atmosphere. The reaction mixture was stirred at 140 ℃ for 24 h. The mixture was cooled down, filtered over celite, washed with DCM, and concentrated. Flash silica gel column chromatography (PE:DCM = 5:1) purification of the residue gave 1.12 g of the isomer **CO5T-10** as yellow oil. ^{1}**H NMR** (400 MHz, $CDCl_3$) δ 7.30 (s, 2H), 7.02 (d, J = 5.4 Hz, 2H), 6.70 (d, J = 5.3 Hz, 2H), 1.61 – 1.49 (m, 4H), 1.33 – 1.09 (m, 32H), 0.95 (ddt, J = 9.0, 6.1, 2.6 Hz, 8H), 0.77 (q, J = 6.9 Hz, 24H). ^{13}**C NMR** (400 MHz, $CDCl_3$) δ 150.84, 135.50, 126.18, 122.51, 121.38, 118.44, 35.83, 35.57, 34.91, 34.78, 29.08, 29.01, 28.98, 28.88, 28.79, 23.06, 14.22, 14.20, 10.92, 10.90, 10.85. **HRMS** (ESI): calcd. for $C_{46}H_{75}O_2S_2Si_2$ $[M+H]^+$: 779.4742, Found: 779.4733.

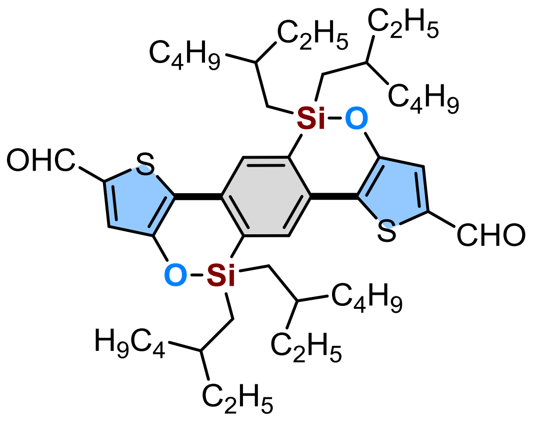

A Vilsmeier reagent, which was prepared with $POCl_3$ (4.1 mL) in DMF (20 mL), was added to a cold solution of the isomer **SiO5T-10** (2.04 g, 2.62 mmol) in dry dichloroethane (50 mL) at 0 ℃ under a nitrogen atmosphere. After being stirred at 100 ℃ for 12 h, the same amount of Vilsmeier reagent was added to the reaction mixture with stirring for additional 12 h. Then, the mixture was poured into ice water (100 mL), neutralized with Na_2CO_3, and then extracted with dichloromethane. The combined organic layer was washed with water and brine, dried over anhydrous $MgSO_4$. After removal of solvent, it was purified by column chromatography on silica gel using petroleum ether/dichloromethane (1:1) as eluent, yielding a yellow solid **11b** (820 mg, 31%). ^{1}**H NMR** (400 MHz, $CDCl_3$) δ 9.82 (s, 2H), 7.50 (s, 2H), 7.36 (s, 2H), 1.62 –

1.42 (m, 4H), 1.37 – 1.07 (m, 32H), 1.02 – 0.91 (m, 8H), 0.83 – 0.60 (m, 24H). **^{13}C NMR** (100 MHz, $CDCl_3$) δ 182.59, 151.51, 137.49, 135.98, 131.86, 129.84, 128.66, 127.59, 35.82, 35.59, 34.92, 34.78, 29.06, 28.97, 28.95, 28.89, 28.77, 23.04, 21.26, 14.19, 10.91, 10.89, 10.86. **MS** (MALDI-TOF): calcd. for $C_{48}H_{75}O_4S_2Si_2$ $[M+H]^+$: 835.4645, Found: 836.80.

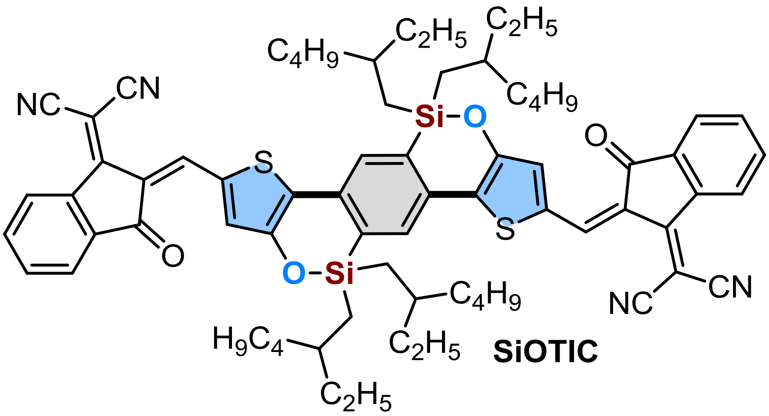

SiOTIC

Compound **11b** (228 mg, 0.27 mmol) and 1,1-dicyanomethylene-3-indanone (210 mg, 1.08 mmol) were dissolved in $CHCl_3$ (40 mL) under a nitrogen atmosphere. 1.1 mL pyridine was added and refluxed for 24 h. After cooling to room temperature, the mixture was poured into water (100 mL) and extracted with $CHCl_3$ (2 × 100 mL). The organic layer was washed with water, and then dried over $MgSO_4$. After removing the solvent, the residue was purified using column chromatography on silica gel employing PE/DCM (1:2) as an eluent, yielding a dark blue solid **SiOTIC** (165 mg, 51%). To ensure the device performance, the chromatographically pure **SiOTIC** was further purified by re-precipitation with MeOH, and dried overnight under vacuum. **^{1}H NMR** (400 MHz, $CDCl_3$) δ 8.76 (s, 2H), 8.72 (d, J = 7.1 Hz, 2H), 7.96 (d, J = 6.9 Hz, 2H), 7.83 – 7.75 (m, 4H), 7.73 (s, 2H), 7.57 (s, 2H), 1.58 (s, 4H), 1.35 – 0.98 (m, 40H), 0.79 (ddd, J = 14.6, 7.4, 4.0 Hz, 24H). **^{13}C NMR** (101 MHz, $CDCl_3$) δ 188.46, 160.30, 152.84, 140.24, 137.49, 137.05, 136.62, 136.31, 135.61, 135.47, 134.78, 133.06, 128.61, 125.57, 123.93, 114.61, 114.48, 70.27, 35.77, 35.50, 34.92, 34.77, 28.99, 28.76, 23.10, 23.09, 23.03, 21.58, 21.50, 21.43, 21.34, 14.25, 14.23, 10.89, 10.87, 10.84. MS (MALDI-TOF): calcd. for C72H83N4O4S2Si2 [M+H]+: 1187.5394, Found: 1189.43.

DFT Calculations

Calculations are performed using Gaussian 03 package. All the electronic structures for the optimized geometry are calculated at B3LYP/6-31G**/B3LYP/6-31+G* level. The long alkyl chain was replaced by ethyl to simplify the calculation.

TG and DSC Curve

TGA was recorded on a Perkin-Elmer Pyris under nitrogen atmosphere at a heating rate of 10 ℃/min. DSC was conducted on a TA Q200 Instrument under nitrogen atmosphere at a heating/cooling rate of 10 ℃/min.

Device Fabrication & Measurements

The OSCs were fabricated with a structure of ITO/PEDOT:PSS/active layer/PNDIT-F3N/Ag. The ITO glass was cleaned by sequential ultrasonic treatment in water, deionized water, acetone and isopropanol, and then treated in an ultraviolet ozone cleaner (Ultraviolet Ozone Cleaner, Jelight Company, USA) for 20 min. The PEDOT:PSS aqueous solution (Baytron P 4083 from H. C. Starck) was filtered through a 0.45mm filter and then spin-coated on precleaned ITO-coated glass at 4000 rpm for 30 s. Subsequently, the PEDOT:PSS film was annealed at 150 ℃ for 20 min in air to form a 30 nm film. A blend solution of donor and acceptor was prepared by dissolving the materials in chlorobenzene, at total solids concentration of 20 mL^{-1}, ratio of D/A is 1:1.2, and then was spin-coated at 2500 rpm onto the PEDOT:PSS layer. Some active layer films were then thermally annealed on a hotplate at 100 ℃ for 10 min. After annealing, the films were naturally cooled to room temperature. Then methanol solution of PNDIT-F3N at a concentration of 0.5 mg mL^{-1} was deposited on the active layer at 3000 rpm for 30 s to afford a cathode buffer layer. Finally, the metal cathode Ag was thermal evaporated under a mask at a base pressure of ~10^{-4}Pa. The photovoltaic area of the device is 4.0mm^{-2}. Optical microscope (Olympus BX51) was used to define the active area of the devices. The J–V characteristics of the OSCs were measured in a nitrogen glove box with a Keithley 2450 Source Measure unit. Oriel Sol3A Class AAA Solar Simulator (model, Newport 94023A) with a 450W xenon lamp and an AM 1.5 filter was used as the light source. The light intensity was calibrated to 100$mWcm^{-2}$ by a Newport Oriel 91,150 V reference cell. The voltage step and delay time were 10 mV and 1 ms, respectively. The scan started from −0.2 V to 1.5 V. The EQE was measured

by Solar Cell Spectral Response Measurement System QE-R3-011 (Enli Technology Co., Ltd., Taiwan). The light intensity at each wavelength was calibrated with a standard single-crystal Si photovoltaic cell. The the relationship of J_{sc} to the light intensity were measured by steady-state current-voltage measurement, the light intensity was modulated by neutral density filters (NDF) with different values of optical density (OD). The mobility of electron was tested by fitting the current-bias characteristics in dark utilizing a field-independent space charge limited current (SCLC) model following the Mott-Gurney law given by $J = \frac{9}{8}\varepsilon_0\varepsilon_r\mu\frac{V^2}{L^3}$. The device structure for hole-only and electron-only device are ITO/PEDOT:PSS/ active layer /MoO_3/Ag and ITO/ZnO/ active layer / PNDIT-F3N/Al, respectively.

Transient photovoltage and photocurrent measurement. The transient photovoltage (TPV) and transient photocurrent (TPC) were performed by performance-all-in-one system(paios).

Photoluminescence measurement. Steady-state photoluminescence (PL) spectra of films were measured with an FLS920 spectro-fluorimeter (Edinburgh Instruments), whose light source system contains 450W xenon lamp and a Glan prism. Spectrograph detection used Ge detector with reponse range from 800 to 1700 nm. The data was collected and analyzed by the connected F900 systems software.

AFM characterization. The film morphology was measured by the atomic Force microscopy (AFM) with the tapping mode from Asylum Reserach. The film samples were prepared followed the fabrication method of the solar cells device.

TEM characterization. Transmission electron microscopy (TEM) images were obtained using a techai F30 instrument at an accelerating voltage of 300 kv. The active layer were spincoated onto PEDOT:PSS. Then the substrate were immersed in the deionized water and the the active layer films were floated. Subsequently, the films were ransferred to a TEM grid.

2. Supplementary Figures

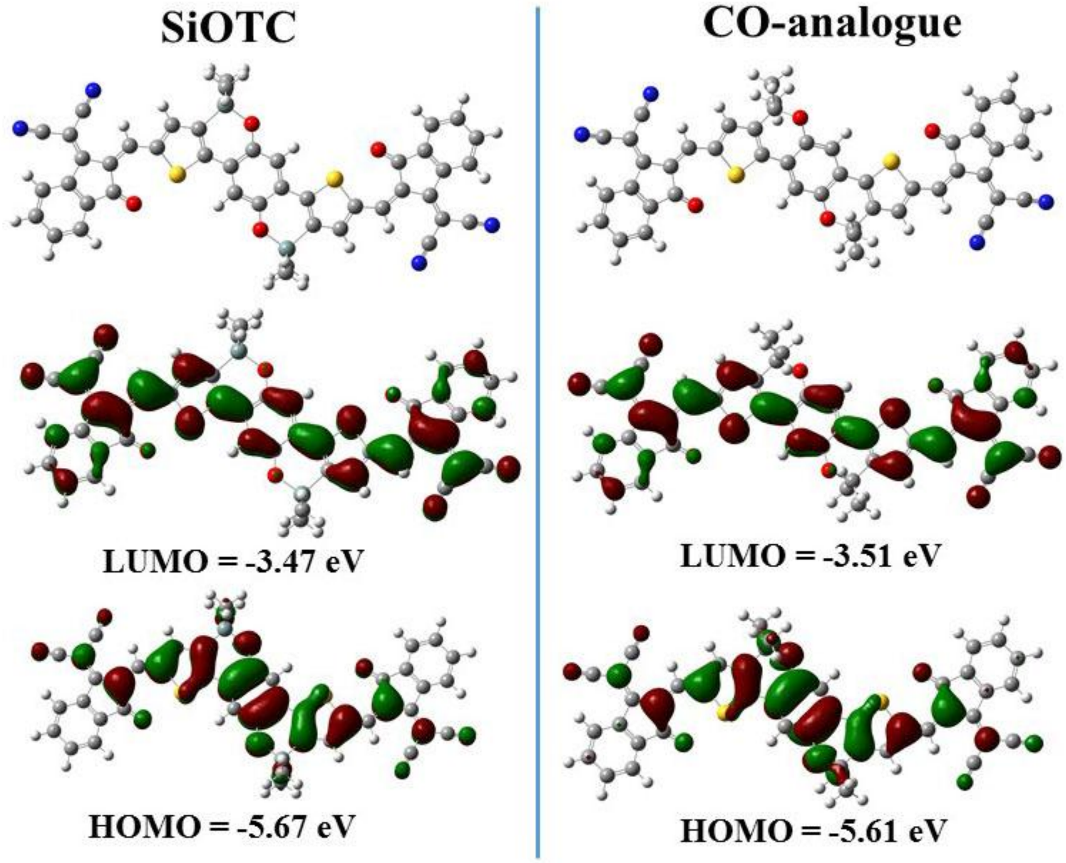

Supplementary Figure 1. Electron distribution of frontier orbitals of **SiOTC** and the carbon-analogue.

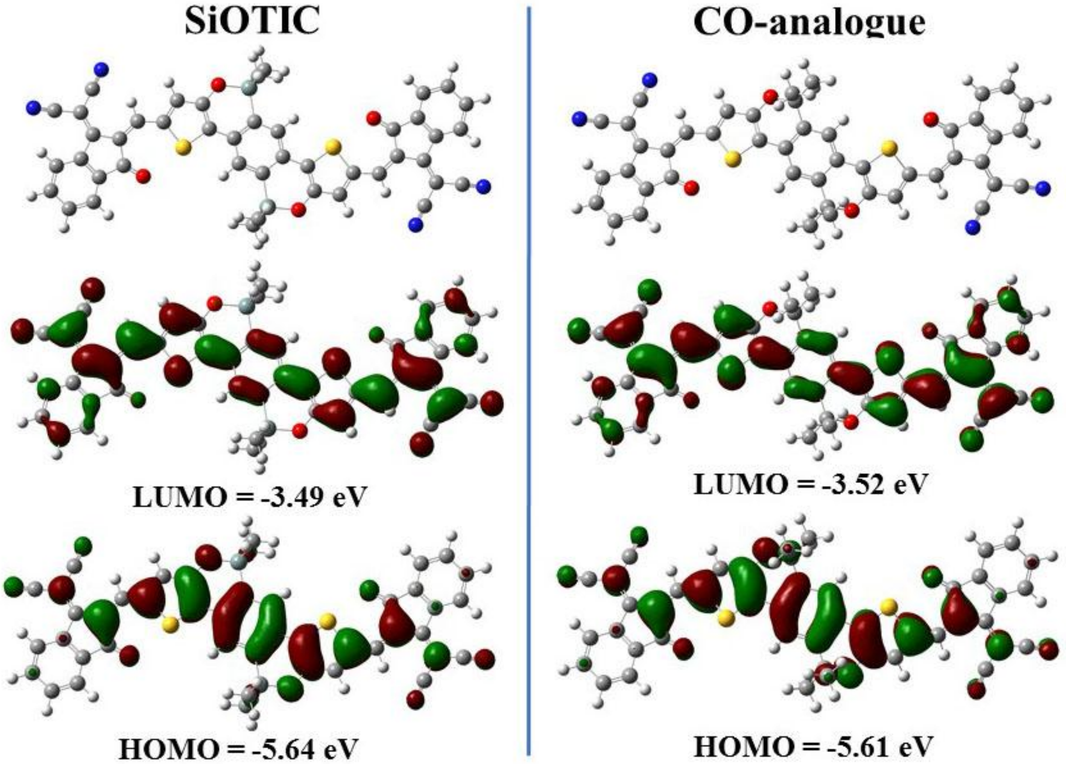

Supplementary Figure 2. Electron distribution of frontier orbitals of **SiOTIC** and the carbon-analogue.

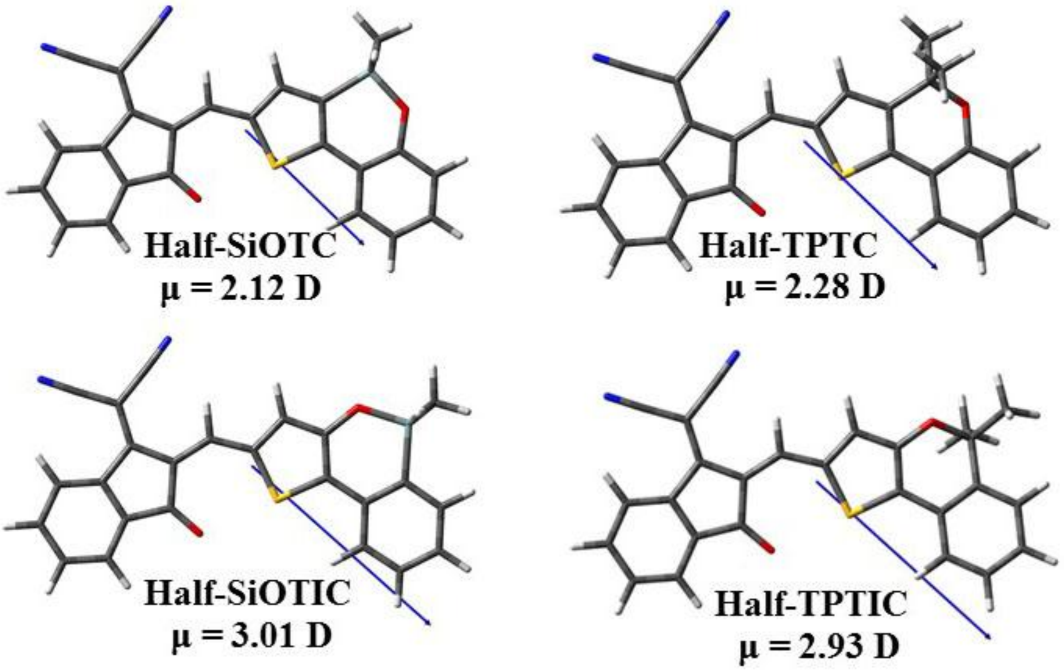

Supplementary Figure 3. The diople moments of the half **SiOTC** and **SiOTIC** as well as their carbon-analogues (**TPTC** and **TPTIC**).

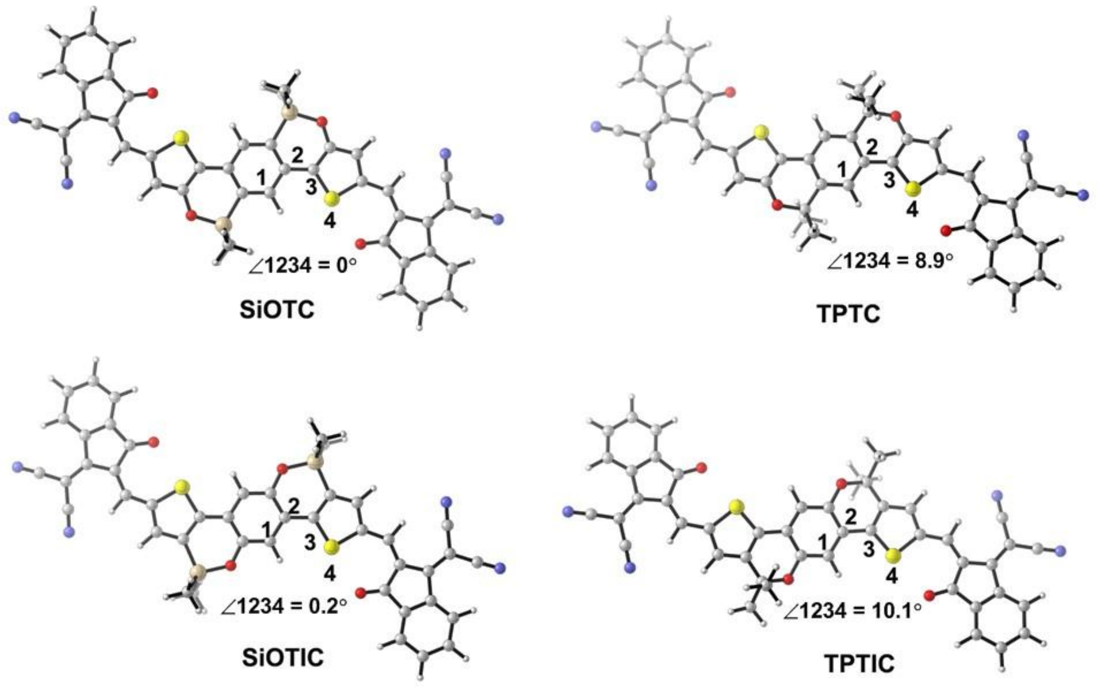

Supplementary Figure 4. The optimal molecular geometries of **SiOTC** and **SiOTIC** as well as their carbon-analogues (**TPTC** and **TPTIC**) by DFT.

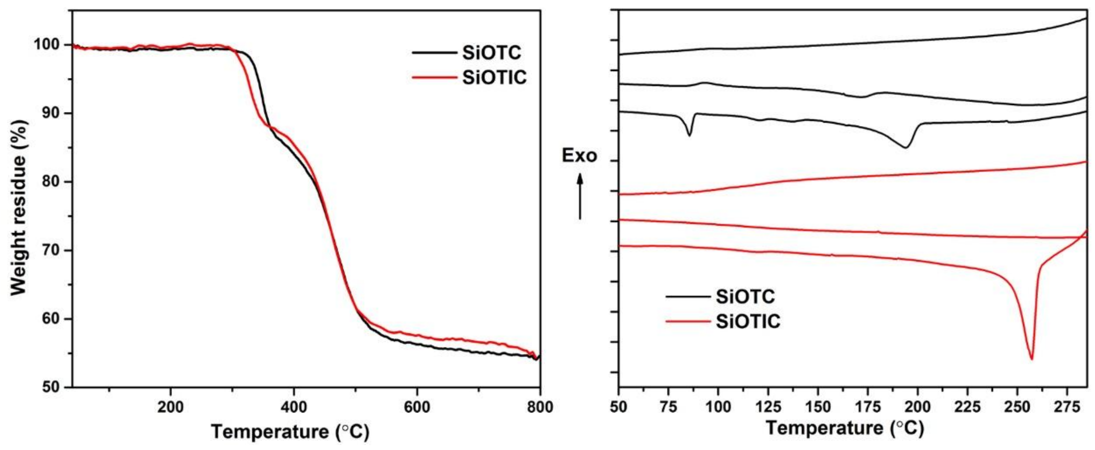

Supplementary Figure 5. TGA measurement of **SiOTC** and **SiOTIC** (left) as well as DSC heating and cooling traces of the **SiOTC** and **SiOTIC** acceptors (right).

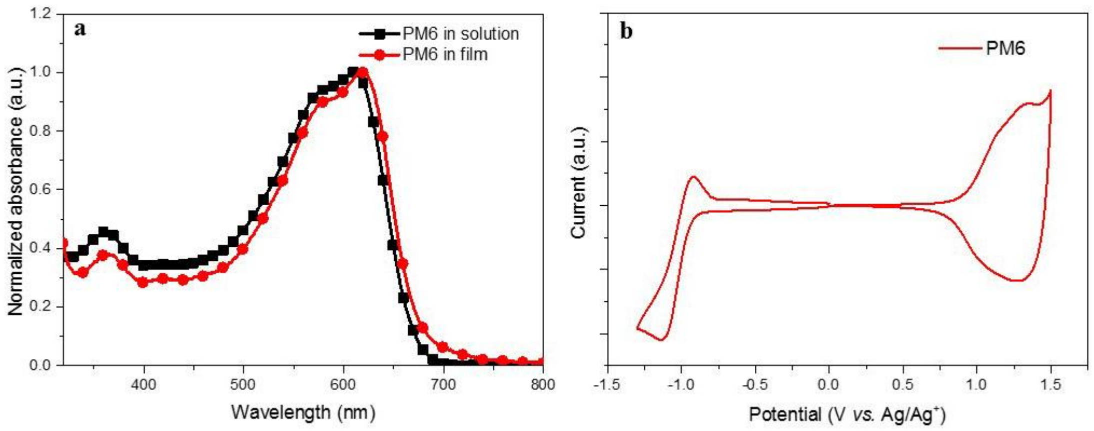

Supplementary Figure 6. Absorption (**a**) and CV profile of the PM6 donor (**b**).

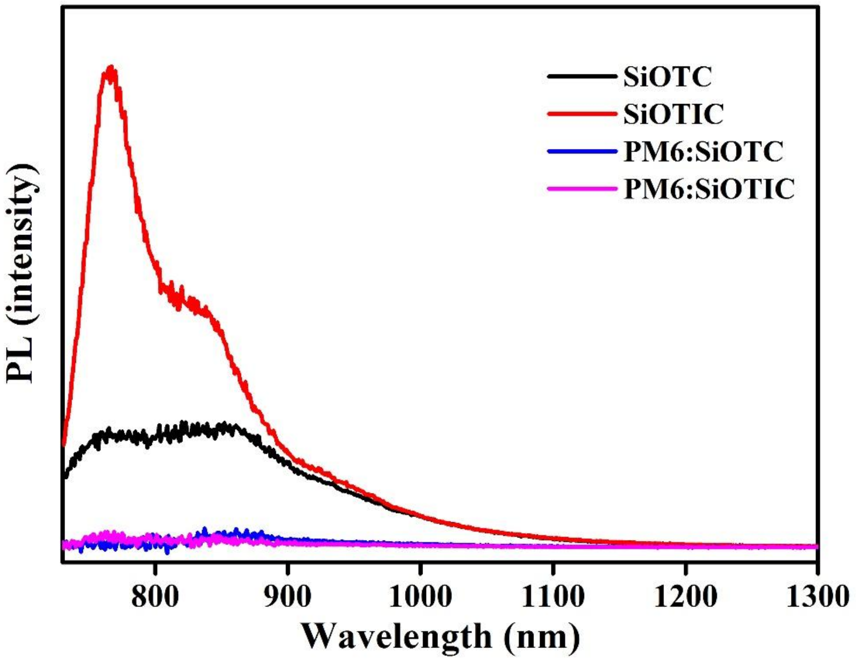

Supplementary Figure 7. Photoluminescence (PL) spectra of the pure **SiOTC** & **SiOTIC** films and the blend films with **PM6**.

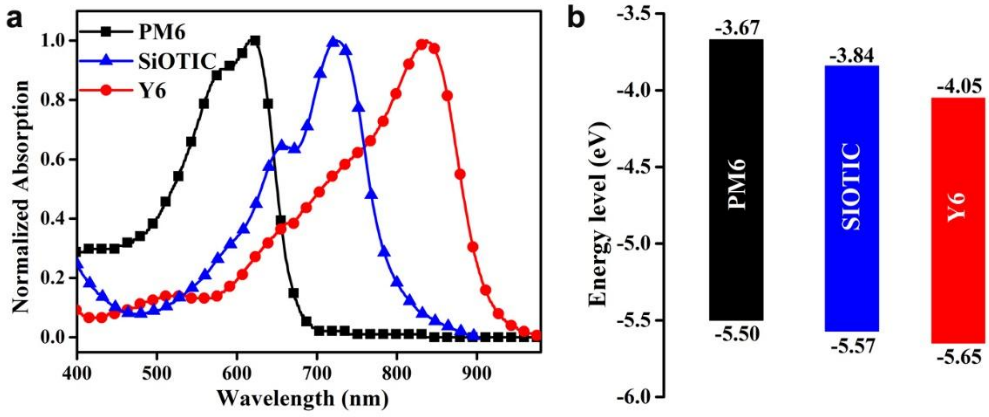

Supplementary Figure 8. (a) Normalized absorption spectra of **PM6**, **SiOTIC** and **Y6** in film and (b) Energy level profiles of **PM6**, **SiOTIC** and **Y6**.

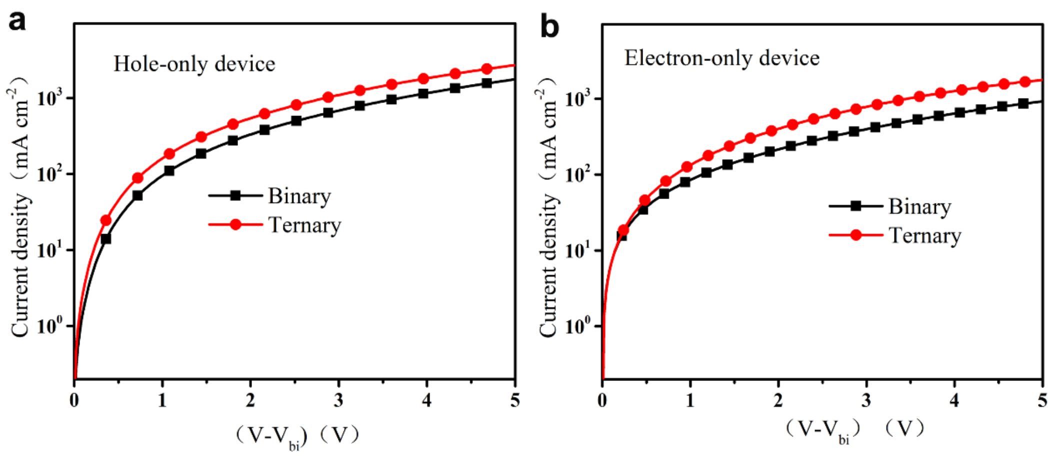

Supplementary Figure 9. The J-V curves of hole-only (a) and electron-only (b) devices based on binary and ternary blend films.

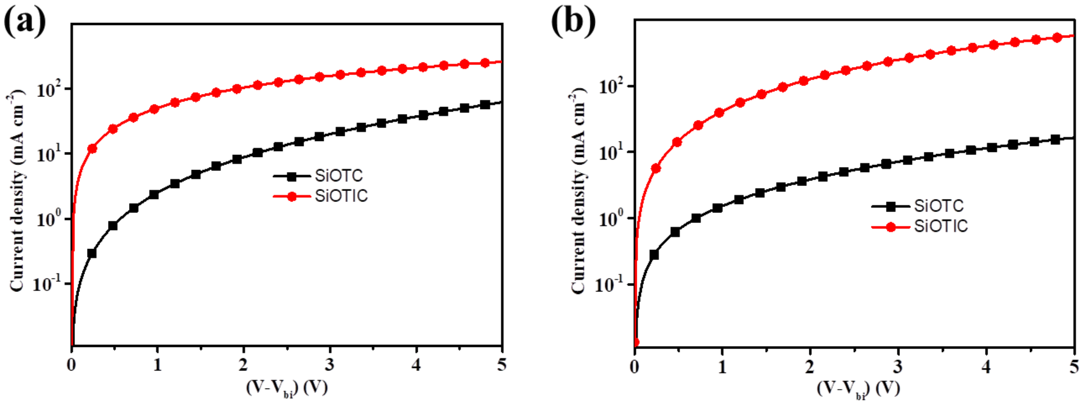

Supplementary Figure 10. Dark J-V curves for the hole-only (a) and (b) electron-only devices.

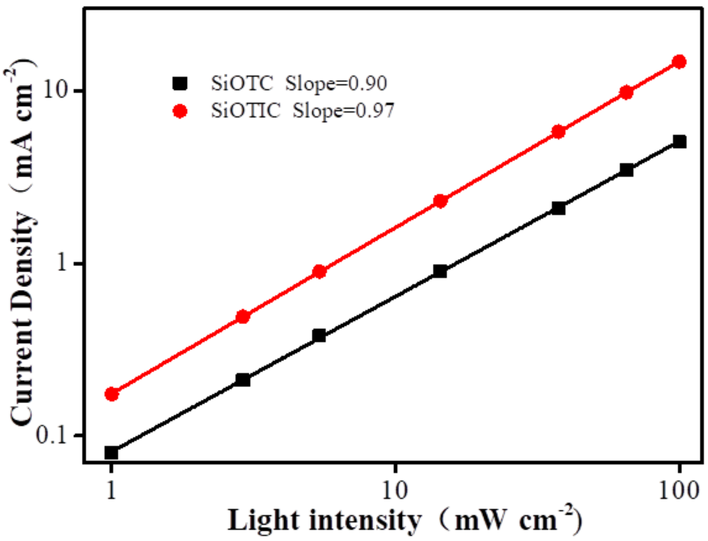

Supplementary Figure 11. J_{sc} vesus light intensity on a double-logarithmic scale for devices based acceptors.

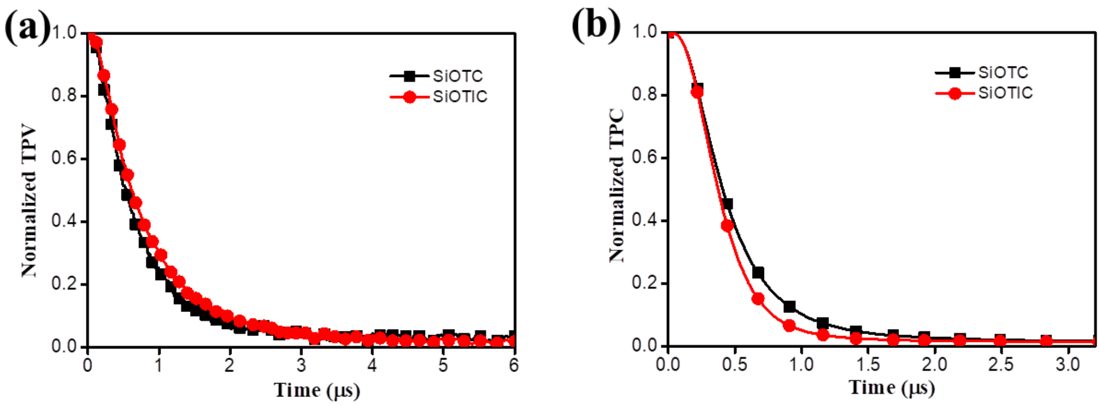

Supplementary Figure 12. (a) the transient photovoltage (TPV) curves and (b) the transient photocurrent (TPC) curves of devices based on **SiOTC** and **SiOTIC**.

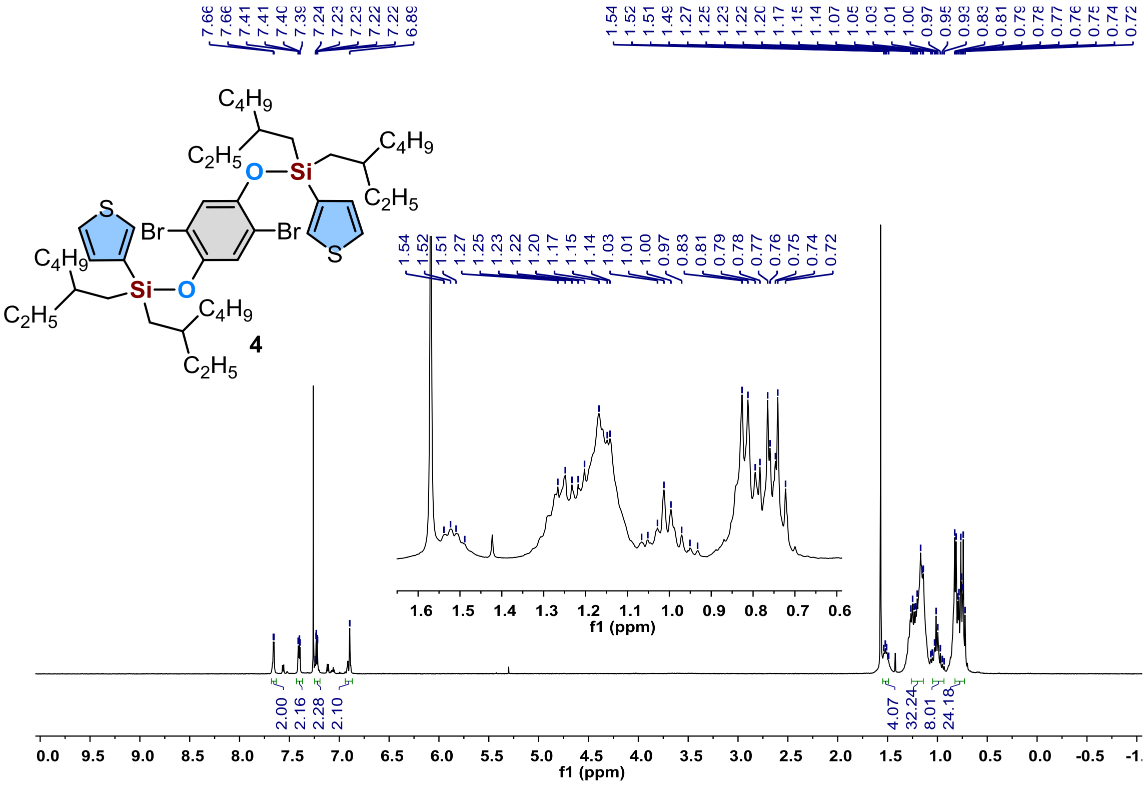

Supplementary Figure 13. ^{1}H NMR (400 MHz, $CDCl_3$) spectrum for **4**

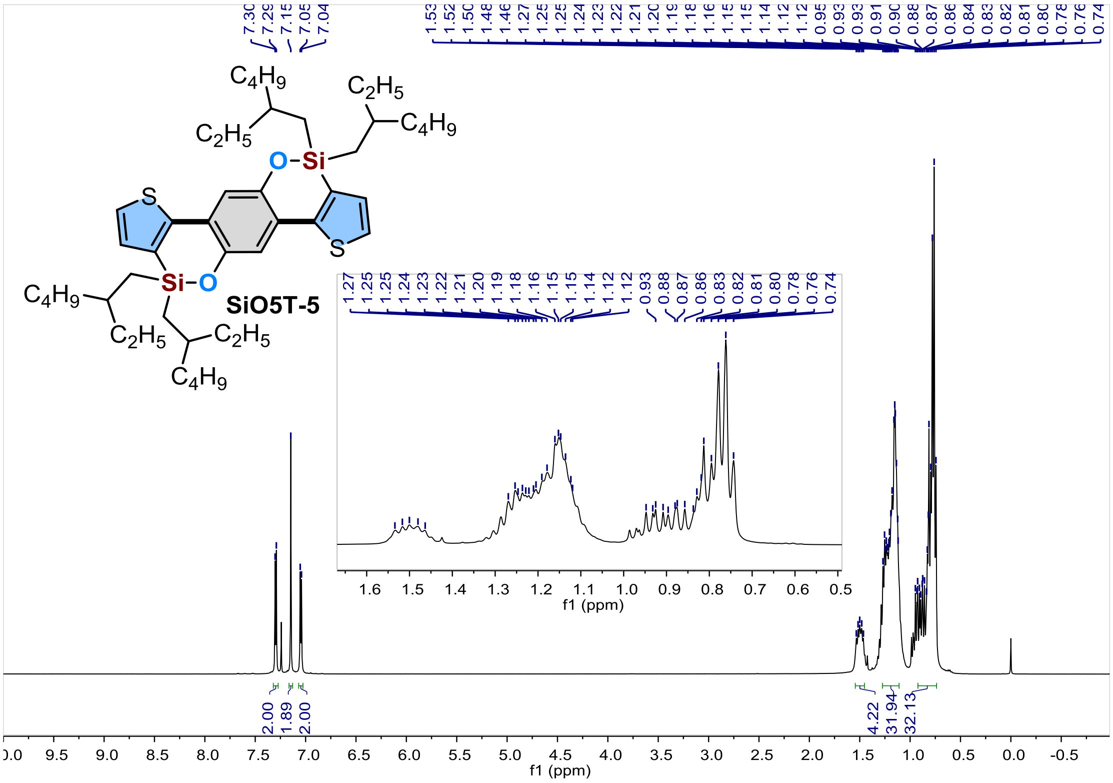

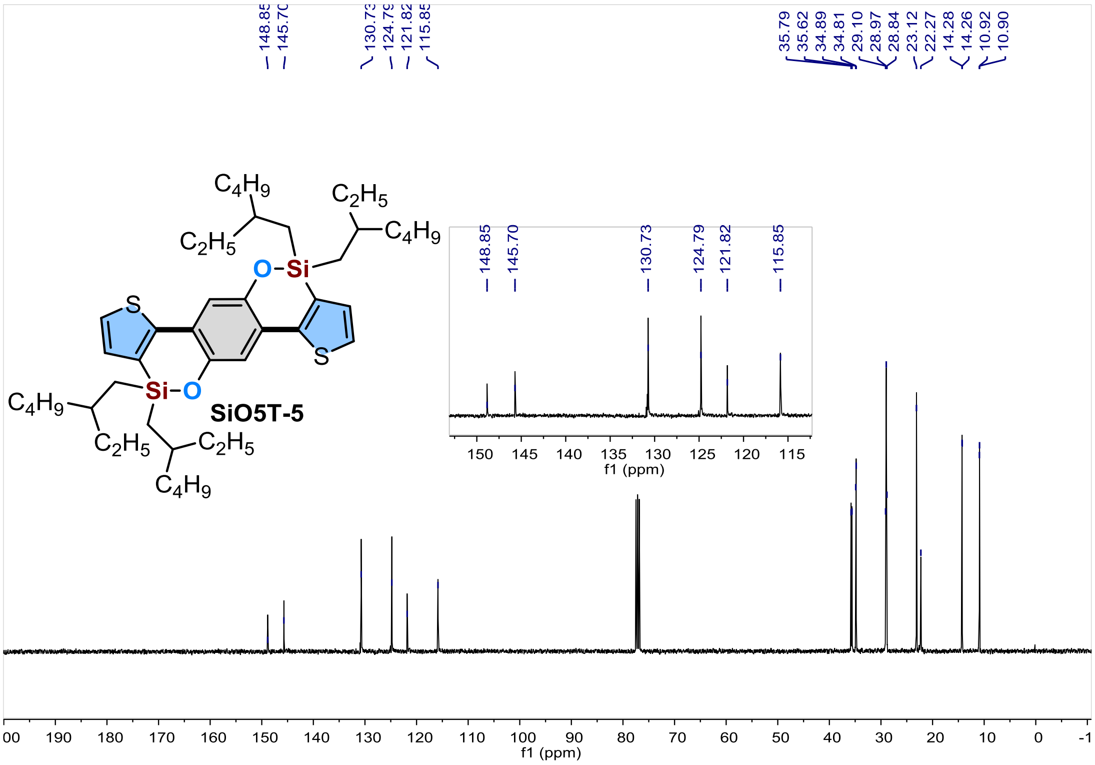

Supplementary Figure 14. ^{1}H NMR (400 MHz, $CDCl_3$) and ^{13}C NMR (100 MHz, $CDCl_3$) spectrum for **SiO5T-5**

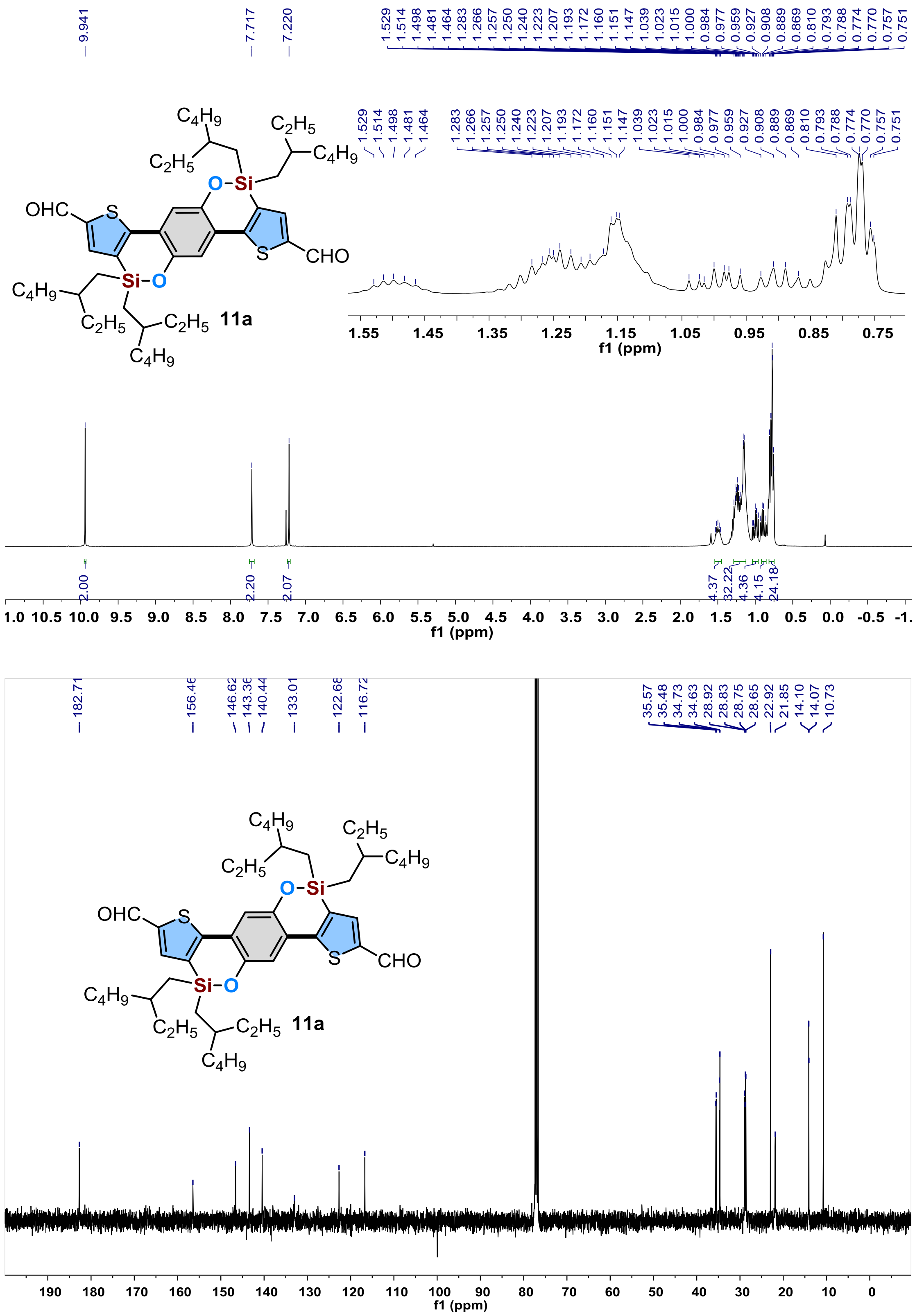

Supplementary Figure 15. ^{1}H NMR (400 MHz, $CDCl_3$) and ^{13}C NMR (100 MHz, $CDCl_3$) spectrum for compound **11a**

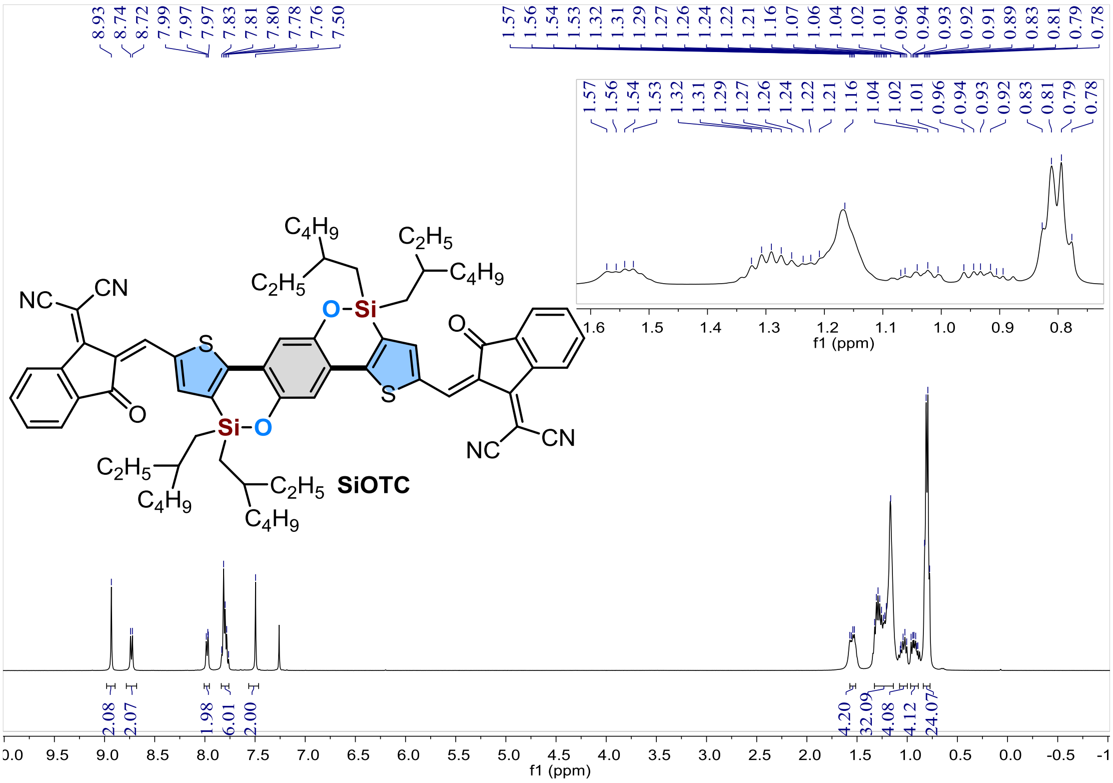

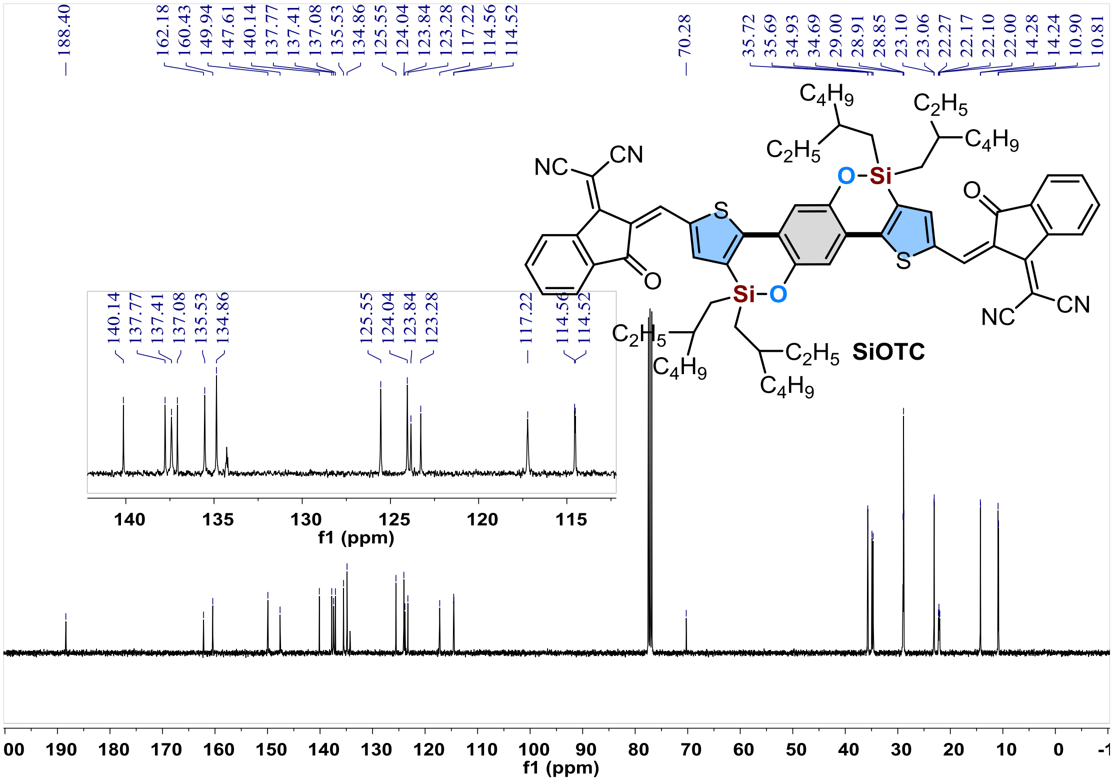

Supplementary Figure 16. ^{1}H NMR (400 MHz, $CDCl_3$) and ^{13}C NMR (100 MHz, $CDCl_3$) spectrum for compound **SiOTC**

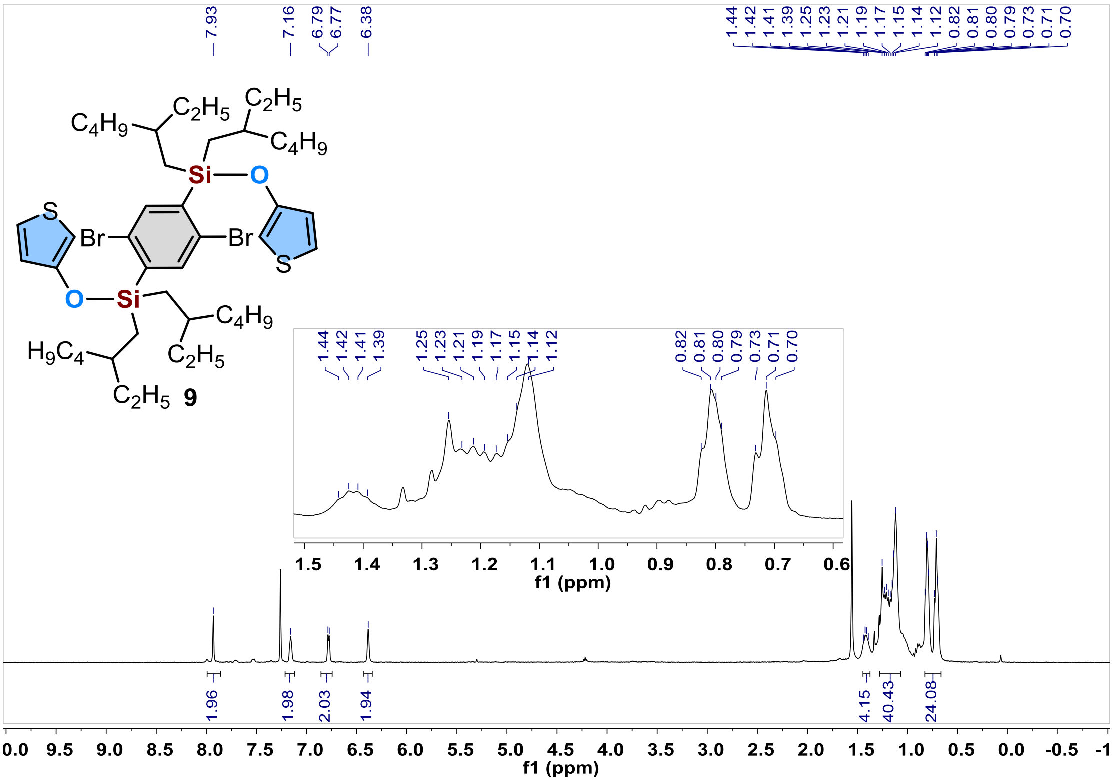

Supplementary Figure 17. ^{1}H NMR (400 MHz, $CDCl_3$) spectrum for compound **9**

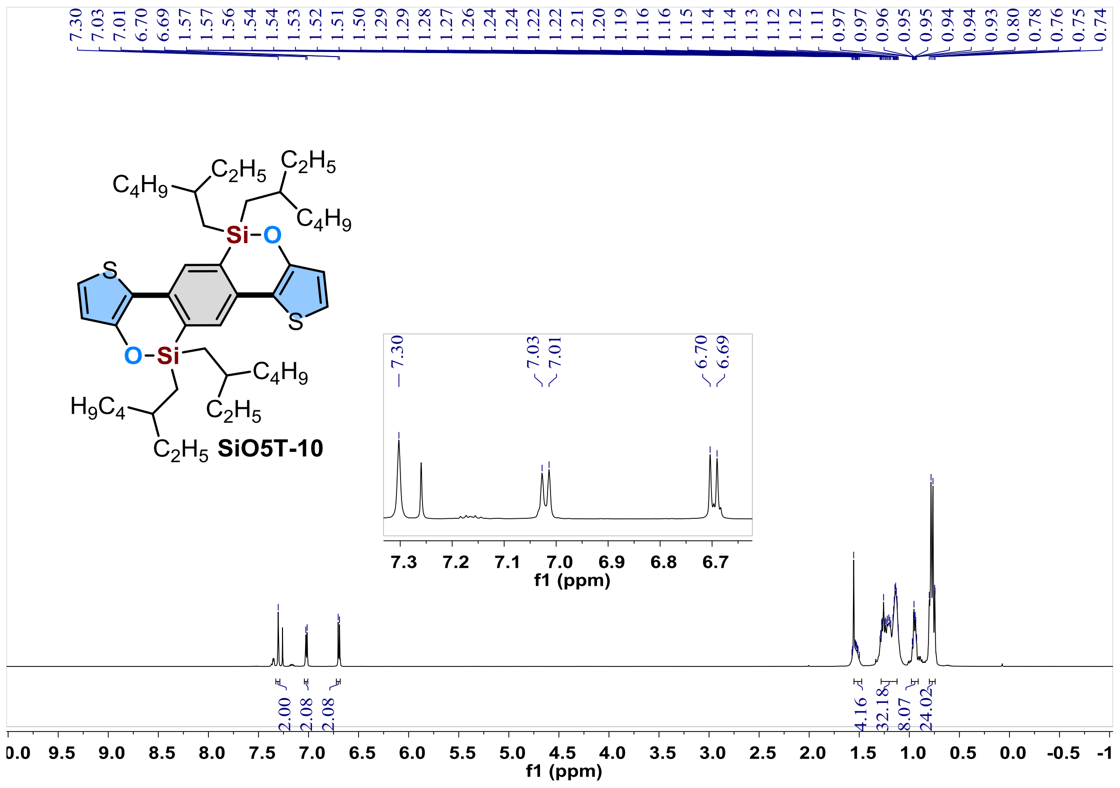

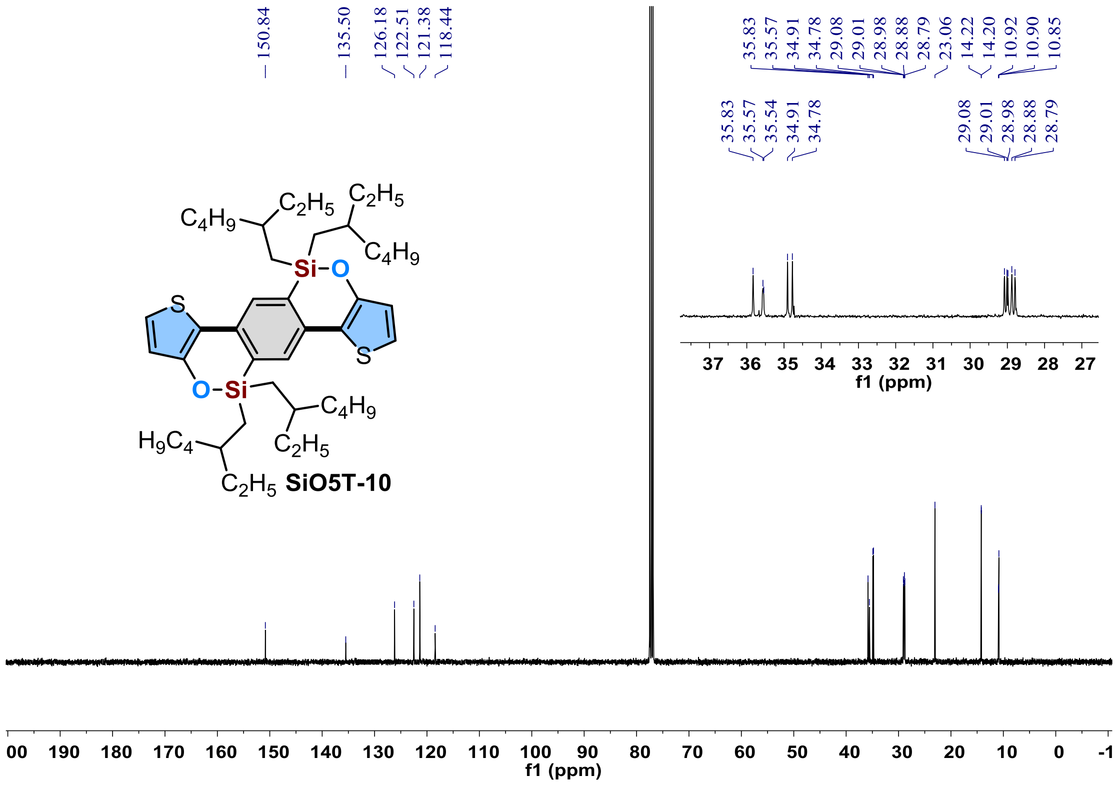

Supplementary Figure 18. ^{1}H NMR (400 MHz, $CDCl_3$) and ^{13}C NMR (100 MHz, $CDCl_3$) spectrum for compound **SiO5T-10**

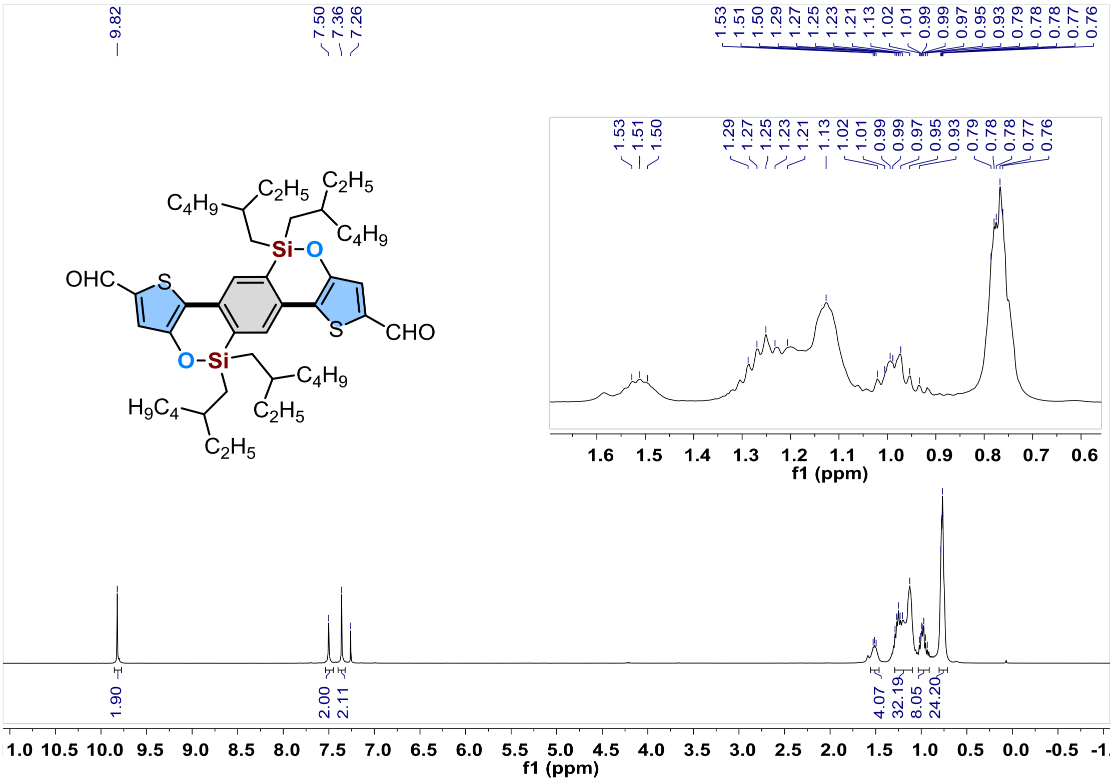

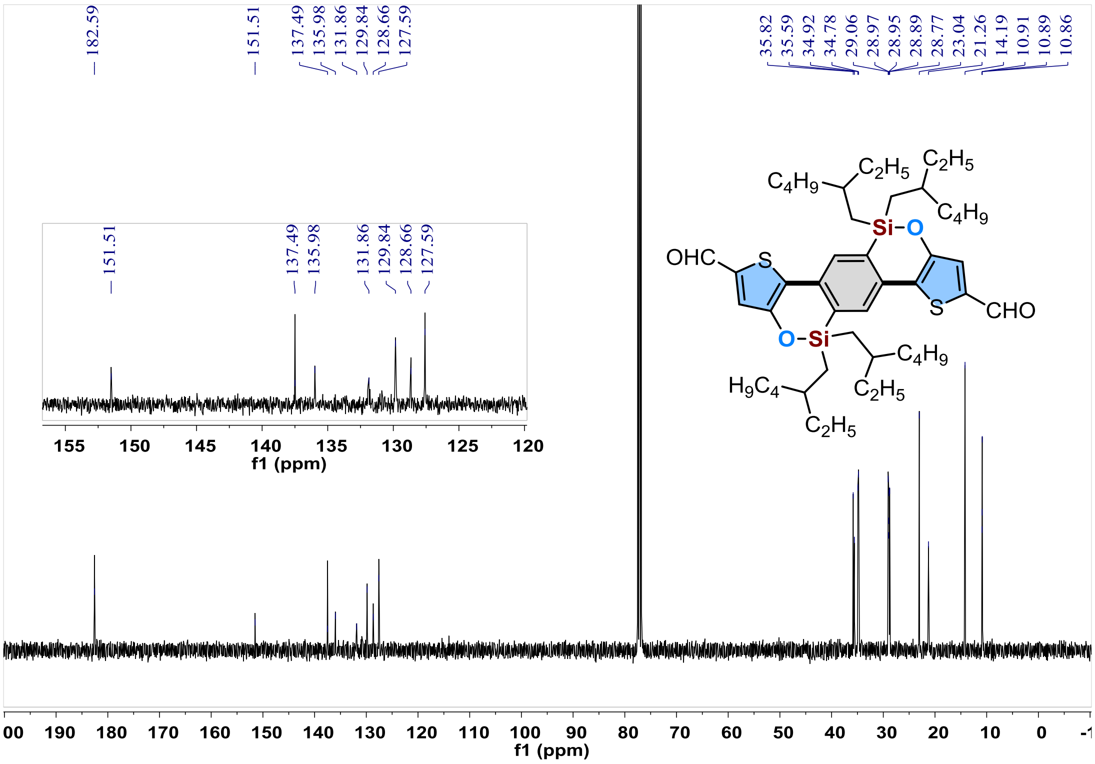

Supplementary Figure 19. ^{1}H NMR (400 MHz, $CDCl_3$) and ^{13}C NMR (100 MHz, $CDCl_3$) spectrum for compound **11b**

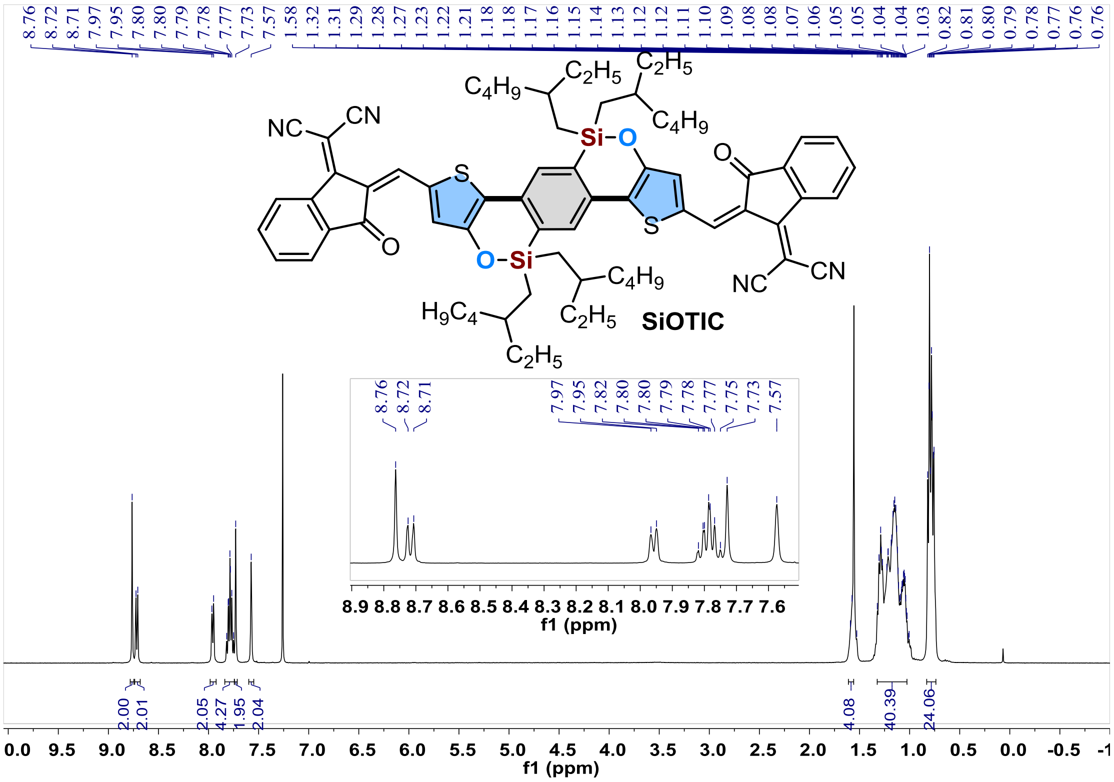

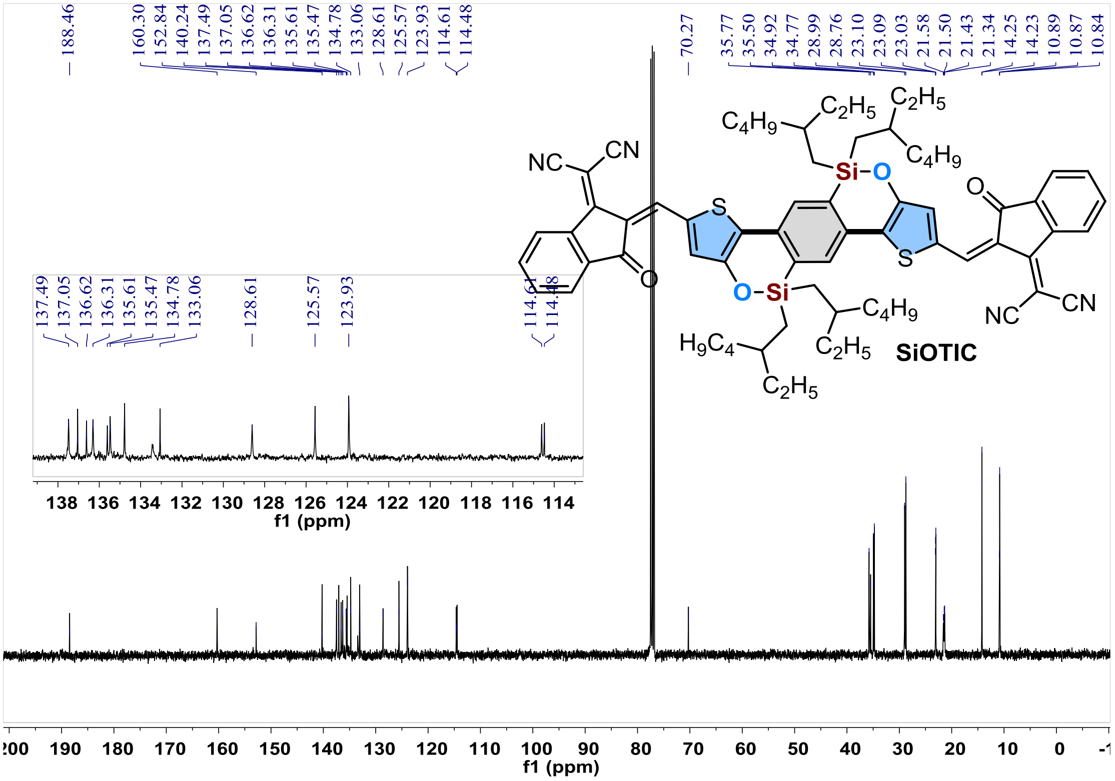

Supplementary Figure 20 ^{1}H NMR (400 MHz, $CDCl_3$) and ^{13}C NMR (100 MHz, $CDCl_3$) spectrum for **SiOTIC**

3. Supplementary Tables

Supplementary Table 1. Extracted mobility of the hole- and electron-only devices based on binary and ternary blend films

Acceptor	Hole Mobility ($cm^2\ v^{-1}\ s^{-1}$)	Electron Mobility ($cm^2\ v^{-1}\ s^{-1}$)	Hole/Electron
Binary(PM6/Y6)	3.2×10^{-4}	2.9×10^{-4}	1.4
Ternary(PM6/SiOTIC/Y6)	5.4×10^{-4}	4.5×10^{-4}	1.2

Supplementary Table 2. Extracted mobility of the hole- and electron-only devices based on acceptors

Acceptor	Hole Mobility ($cm^2\ v^{-1}\ s^{-1}$)	Electron Mobility ($cm^2\ v^{-1}\ s^{-1}$)	Hole/Electron
SiOTC	8.5×10^{-6}	5.3×10^{-6}	1.6
SiOTIC	1.7×10^{-4}	1.3×10^{-4}	1.3

Supplementary Table 3. Extracted lifetime and sweeping out time of carriers in devices

Device	τ(μs)	t_s (μs)	τ/t_s
SiOTC	0.60	0.44	1.4
SiOTIC	0.74	0.37	2.0

Supplementary Table 4. The peak positions, d-spacing as well as coherence lengths of GIWAXS

	Out-of-Plane						In-Plane								
	π-π stacking cell axis (010)			lamellar stacking cell axis (100)			π-π stacking cell axis (010)			lamellar stacking cell axis (100)			Backbone stacking (001)		
	q (Å^{-1})	d-spacing (Å)	Coherence length (Å)	q (Å^{-1})	d-spacing (Å)	Coherence length (Å)	q (Å^{-1})	d-spacing (Å)	Coherence length (Å)	q (Å^{-1})	d-spacing (Å)	Coherence length (Å)	q (Å^{-1})	d-spacing (Å)	Coherence length (Å)
SiOTC	1.774	3.541	69.158	N/A			N/A			0.288	21.758	119.998	0.366	17.146	208.238
SiOTIC	1.813	3.466	58.714	N/A			N/A			N/A			0.398	15.781	307.186
SiOTC:PM6	1.760	3.569	53.058	0.300	20.961	81.795	N/A			0.299	20.994	134.976	0.365	17.234	223.717
SiOTIC:PM6	1.815	3.461	70.139	0.307	20.463	81.996	N/A			0.296	21.223	139.261	0.398	15.777	279.564

4. Supplementary References

[1]. C. Huang, and V. Gevorgyan. *J. Am. Chem. Soc.* **2009**, *131*, 10844.

[2]. S. Zhang, J. Gao, W. Wang, C. Zhan, S. Xiao, Z. Shi, and W. You. *ACS Appl. Energy Mater.* **2018**, *1*, 1276.

[3]. Y. Li, Z. Li, C. Wang, H. Li, H. Lu, B. Xu, and W. Tian. *J. Polym. Sci. Pol.* **2010**, *48*, 2765.

[4]. X. Wang, F. Zhang, J. Gao, Y. Fu, W. Zhao, R. Tang, W. Zhang, X. Zhuang, and X. Feng. *J. Org. Chem.* **2015**, *80*, 10127.

[5]. T. Hafner, A. Torvisco, and F. Uhlig. *J. Organomet. Chem.* **2018**, *875*, 1.
