# Supplementary material for: Silicon and oxygen synergistic effects for the discovery of new high-performance nonfullerene acceptors
Source: Nat Commun. 2020 Nov 16;11:5814. doi: 10.1038/s41467-020-19605-z (PMC7669892; doi:10.1038/s41467-020-19605-z)
Supplement: Supplementary file 3 — Solar Cells Reporting Summary [file 41467_2020_19605_MOESM3_ESM.pdf]

nature research

Corresponding author(s): Dongbing Zhao

Solar Cells Reporting Summary

Nature Research wishes to improve the reproducibility of the work that we publish. This form is intended for publication with all accepted papers reporting the characterization of photovoltaic devices and provides structure for consistency and transparency in reporting. Some list items might not apply to an individual manuscript, but all fields must be completed for clarity.

For further information on Nature Research policies, including our data availability policy, see Authors & Referees.

ü Experimental design

Please check: are the following details reported in the manuscript?

1. Dimensions

Item	Yes	No	Details
Area of the tested solar cells	x		Described in "Device Fabrication & Measurements of Supplementary Information".
Method used to determine the device area	x		Described in "Device Fabrication & Measurements of Supplementary Information".

2. Current-voltage characterization

Item	Yes	No	Details
Current density-voltage (J-V) plots in both forward and backward direction		x	J-V plot in forward direction since there is no hysteresis in polymer solar cells.
Voltage scan conditions<br>*For instance: scan direction, speed, dwell times*	x		Described in "Device Fabrication & Measurements of Supplementary Information".
Test environment<br>*For instance: characterization temperature, in air or in glove box*	x		Described in "Device Fabrication & Measurements of Supplementary Information".
Protocol for preconditioning of the device before its characterization		x	No preconditioning protocol.
Stability of the J-V characteristic<br>*Verified with time evolution of the maximum power point or with the photocurrent at maximum power point; see ref. 7 for details.*		x	No.

3. Hysteresis or any other unusual behaviour

Item	Yes	No	Details
Description of the unusual behaviour observed during the characterization		x	Polymer solar cells do not have hysteresis problems.
Related experimental data		x	No.

4. Efficiency

Item	Yes	No	Details
External quantum efficiency (EQE) or incident photons to current efficiency (IPCE)	x		Provided in Figures 2b and 2d.
A comparison between the integrated response under the standard reference spectrum and the response measure under the simulator	x		Provided in Table 2.
For tandem solar cells, the bias illumination and bias voltage used for each subcell		x	No tandem solar cells are fabricated in this study.

5. Calibration

Item	Yes	No	Details
Light source and reference cell or sensor used for the characterization	x		Described in "Device Fabrication & Measurements of Supplementary Information".
Confirmation that the reference cell was calibrated and certified	x		Described in "Device Fabrication & Measurements of Supplementary Information".

Calculation of spectral mismatch between the reference cell and the devices under test

- [x] Yes
- [ ] No

Described in "Device Fabrication & Measurements of Supplementary Information".

6. Mask/aperture

Size of the mask/aperture used during testing

- [x] Yes
- [ ] No

Described in "Device Fabrication & Measurements of Supplementary Information".

Variation of the measured short-circuit current density with the mask/aperture area

- [ ] Yes
- [x] No

The device was only measured by the mask with 4.00 cm2 area.

7. Performance certification

Identity of the independent certification laboratory that confirmed the photovoltaic performance

- [ ] Yes
- [x] No

We focus on design of new acceptors, the efficiency values were not certified.

A copy of any certificate(s)
Provide in Supplementary Information

- [ ] Yes
- [x] No

No certification.

8. Statistics

Number of solar cells tested

- [x] Yes
- [ ] No

Provided in Table 2.

Statistical analysis of the device performance

- [x] Yes
- [ ] No

Provided in Table 2.

9. Long-term stability analysis

Type of analysis, bias conditions and environmental conditions
For instance: illumination type, temperature, atmosphere humidity, encapsulation method, preconditioning temperature

- [ ] Yes
- [x] No

No.
